# Comparative analysis of PI and fuzzy logic controller for grid connected wind turbine under normal and fault conditions

**DOI:** 10.1038/s41598-024-85073-w

**Published:** 2025-01-14

**Authors:** Mohamed Bahgat, Mohamed Ezzat, Mahmoud A. Attia, S. F. Mekhamer, Nourhan M. Elbehairy

**Affiliations:** 1 Electrical Power and Machines Department, Ain Shams University, Cairo, Egypt; 2 Electrical Engineering Department, Future University in Egypt, New Cairo, Egypt; 3 Electrical Power and Machines Department, Egyptian Chinese University, Cairo, Egypt

**Keywords:** Doubly fed induction generator, Fuzzy logic controller, Proportional integral controller, Electrical and electronic engineering, Energy grids and networks

## Abstract

This research is dedicated to improving the control system of wind turbines (WT) to ensure optimal efficiency and rapid responsiveness. To achieve this, the fuzzy logic control (FLC) method is implemented to control the converter in the rotor side (RSC) of a doubly fed induction generator (DFIG) and its performance is compared with an optimized proportional integral (PI) controller. The study demonstrated an enhancement in the performance of the DFIG through the utilization of the proposed FLC, effectively overcoming limitations and deficiencies observed in the conventional controllers, this approach significantly improved the performance of the wind turbine. Additionally, the selected membership functions were found to be highly compatible with the unique characteristics of wind energy. The optimization process is implemented for the controllers of both the grid side converter (GSC) and RSC. Through simulated analyses conducted using MATLAB/Simulink software, comprehensive assessments are carried out. The robustness of the FLC is evaluated compared to the optimized controllers across various wind profiles and challenging fault conditions. The results demonstrate satisfactory performance of the FLC in terms of steady-state time, stability, and precision under diverse wind speed profiles. The FLC achieves a significantly better settling time than the enhanced PI, improving by approximately 14–70% under normal conditions and 40–70% under various fault conditions. Additionally, the FLC outperforms the enhanced PI in fault conditions by reducing peak-to-peak oscillations by about 30–65%. It also delivers a smaller steady-state error, with improvements of around 2–4% under both normal conditions and most fault scenarios.

## Introduction

The energy consumption increases enormously because of the huge increase in population and industry; as a result, the power generation map should be changed by the replacement of the conventional generations with renewable ones^1,2^. The majority of research in this field has focused on wind and solar farms, as well as ways to improve their grid interface^3^. Wind energy is the most favorable renewable energy source and has a great potential to be a major player in the electrical power generation map due to its economic viability, environmental friendliness, and inexhaustibility. In fact, employing wind energy respects the environment and aids in avoiding the generation of unwanted carbon dioxide^4–6^. The development of wind turbine technology has increased the energy output, reduced costs, and boosted the deployment of wind turbines in both offshore and onshore facilities^7,8^.

The wind generating capacity added globally in 2023 was 117 GW, increasing the generation capacity of wind power to pass 1 TW demonstrating 13% yearly growth^9^. By 2050, It is anticipated that wind power will have a significant impact on the global energy infrastructure, producing more electricity than any other source with eight thousand GW installed capacity^1,9^.

Two types of wind power generators are variable-speed wind turbines (VSWTs) and fixed-speed wind turbines (FSWTs)^10,11^. Variable speed wind turbines (VSWT) have recently taken the place of fixed speed wind turbines. WT can indeed extract the most power from the wind when it is working at a variable speed. DFIG, squirrel cage induction generator (SCIG), wound field synchronous generator (WFSG), and permanent magnet synchronous generator (PMSG) are the types of generators that may be utilized with variable speed wind turbines^6,12,13^. Due to the DFIG’s effectiveness, minimal power losses, simplicity of operation, reduced cost, very little maintenance, and it functions within a speed range of ± 30% of the synchronization speeds, ensuring a decrease in the size of the static converters, which are linked between the DFIG rotor winding and the grid^14^. It has gained widespread application^15^. The integration of large wind power generators into current grids has brought various challenges related to efficiency, reliability, and grid integration. This has prompted researchers to propose and develop a variety of control systems that can generate high-quality wind energy^16^. The most used control technique is the PI controller because of its effectiveness under normal conditions and simplicity of usage^15,17^. However, the PI controller-based control techniques don’t provide a good performance in terms of accuracy when faced with difficult operating situations such as parameter changes and impact of wind disturbance^18^. The gains of the PI controller have been adjusted using a variety of optimization techniques^15–18^. In^16^, the gains of a fractional order PI controller are fine-tuned using the genetic algorithm, which lowers steady-state error and speeds up the settling time. In^19^, the writers suggested an intelligent PI controller that improved response time and reduced error through adaptive particle swarm optimization. In^20^, the weighting matrices ensuring optimal performance at various wind speeds are determined using the whale optimization process. Gains are approximated in^21^, pole compensation is used to compute gains, and Particle Swarm Optimization is used to build the RSC’s control^1^. In^22^&^23^, the writers discussed the robust control of the DFIG with an energy storage system using maximum power point tracking (MPPT), where in^24^, the energy storage capacity estimation accuracy was enhanced using the bald eagle search algorithm. In^25^, the authors discussed the enhancement of the power parameters using dual-layered tilt fuzzy control. In^26^, the authors demonstrated the Improvement in Low Voltage Ride Through (LVRT) capability of DFIG wind turbine through an enhanced protection system using an advanced nonlinear control loop. In^27^, the writers studied improving the ability of DFIG to ride through faults by employing adaptive backstepping commands and parametric estimation within the nonlinear forward power controller design. In^28^, the authors worked on the optimization of the direct power control of DFIG using super twisting algorithm.

In recent years, many researchers focused on fuzzy logic control and its improvement. Genetic algorithm is used to improve the FLC installed in the RSC, system is validated by studying the system under different wind speeds and three phase faults^29^. An interval type-2 fuzzy logic controller applied on a DFIG in the case of disturbance in the wind speed and in case of changing the system parameters^6^. Fuzzy logic control was utilized to enhance the efficiency of direct power control technique and direct voltage control technique under several transient conditions^30^. FLC was used in vector control of DFIG-GSC, vector control of DFIG-RSC, control DC bus voltage and max power point tracking strategy^31^. Different real time controllers are used with DFIG to enhance the total harmonic distortion and reduce the fluctuation in its power output as in^32^&^33^.

In this paper, the performance of the DFIG was improved by the proposed FLC that effectively addressed limitations and deficiencies observed in prior research, which focused on improving the performance of the WT using conventional controllers not a real time optimization controller. Moreover, the proposed membership functions were deemed well-suited for the characteristics of wind energy.

The following is how the paper is set up: System model details are provided in Section II. The control system is described in Section III. The various controllers are illustrated in Section IV. In Section V, the simulation is covered. In Section VI, the robustness test is shown. Section VII comes to its results at the end.

## The system model

**Fig. 1:**
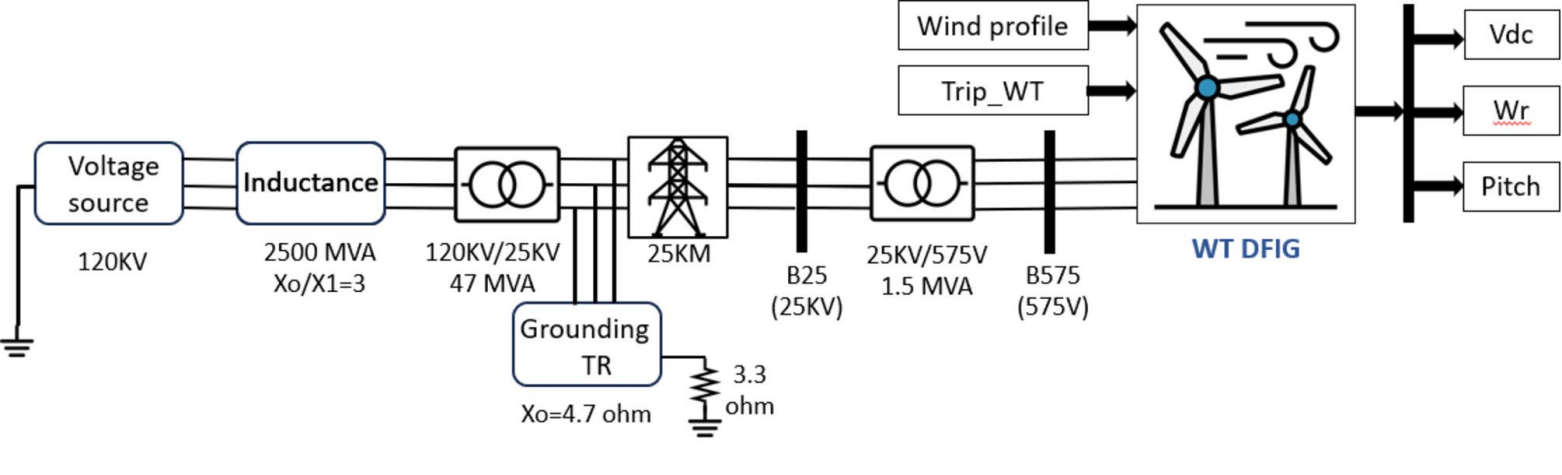
MATLAB/Simulink model of the system.

Figure 1depicts the MATLAB model of the system under study^34^. The component of the system is a 9 MW wind farm connected to the grid with six VSWTs that are each 1.5 MW in size. Two groups of power transformers are used to transmit electricity for a 120 kV grid. The first group, which consists of six transformers with a two MVA capacity each, steps up the voltage from 575 V to 25 kV before transmitting it over a 25-kilometer transmission line to the grid’s 120 kV level using 47 MVA stepping transformer. Table 1contains a list of the system parameters^1^.

**Table 1:** System parameters.

Parameters	Values
Rated power	1.5 MW
Frequency	60 HZ
line-to-line voltage	575 V
Stator inductance	0.171 PU
Stator resistance	0.00706 PU
Rotor inductance	0.156 PU
Rotor resistance	0.005 PU
Mutual inductance	2.9 PU
Number of pole pairs	3
Constant of Inertia	0.74 S

**Fig. 2:**
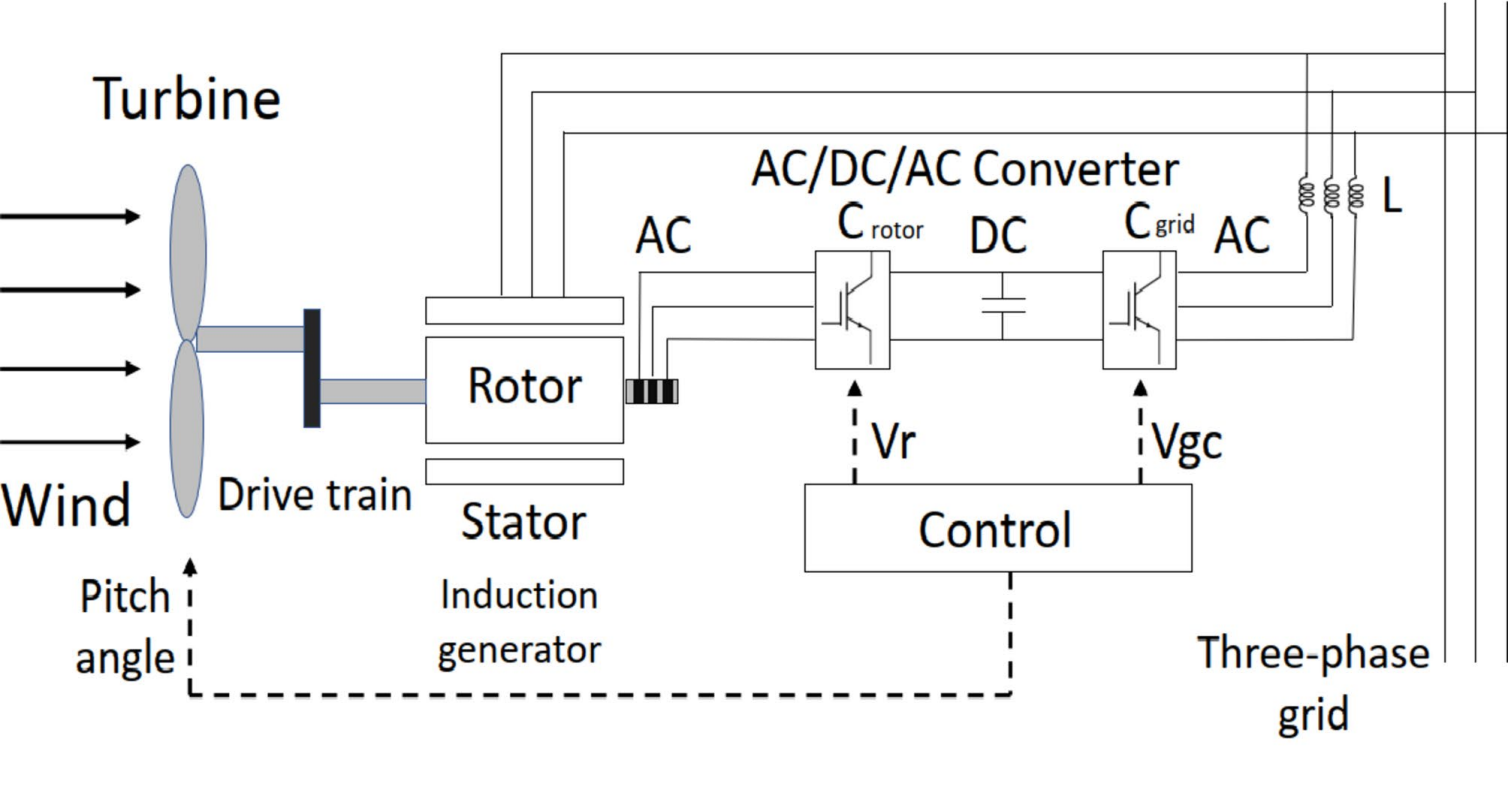
WTDFIG components.

Figure 2displays the elements of the WTDFIG system^35^. This includes the grid-side converter, connected to the grid, and the converter in rotor side, linked to the three-phase rotor winding. The three-phase stator winding is connected to the grid, and a capacitor on the DC side serves as a DC voltage source.

The Kinetic power of the wind is transformed to mechanic power where the induction generator’s rotor and stator windings transfer the mechanical energy extracted by the wind turbine into electrical energy^36^, which is then transmitted to the grid. The control system in place generates the command signals of pitch angle and voltage to control the power output of the wind turbine, DC bus voltage, and the grid terminal’s output voltage or reactive power. This information is derived from reference^37^.

## Control system

A. Rotor-Side Converter Controller.

1. Active Power Control.

**Fig. 3:**
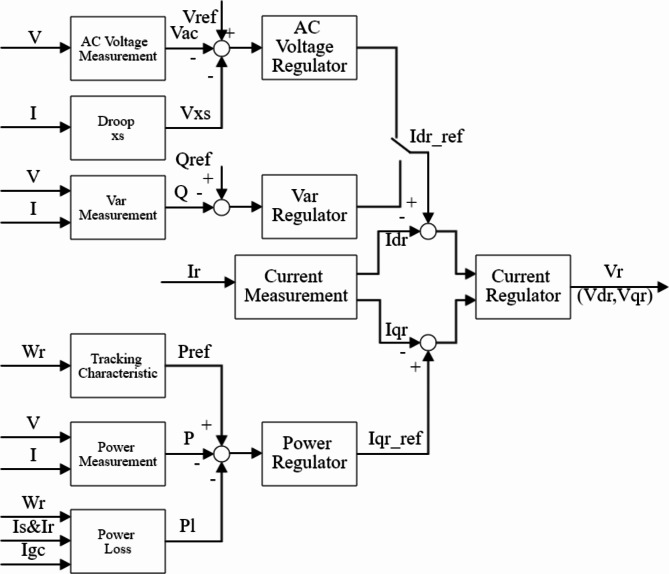
Rotor-side converter controller.

Figure 3illustrates the constructed control loop^37^, utilizing a PI controller to remove any power discrepancies. The regulation of power involves monitoring a specific power-speed relationship.

2) Voltage and Reactive Power Control.

**Fig. 4:**
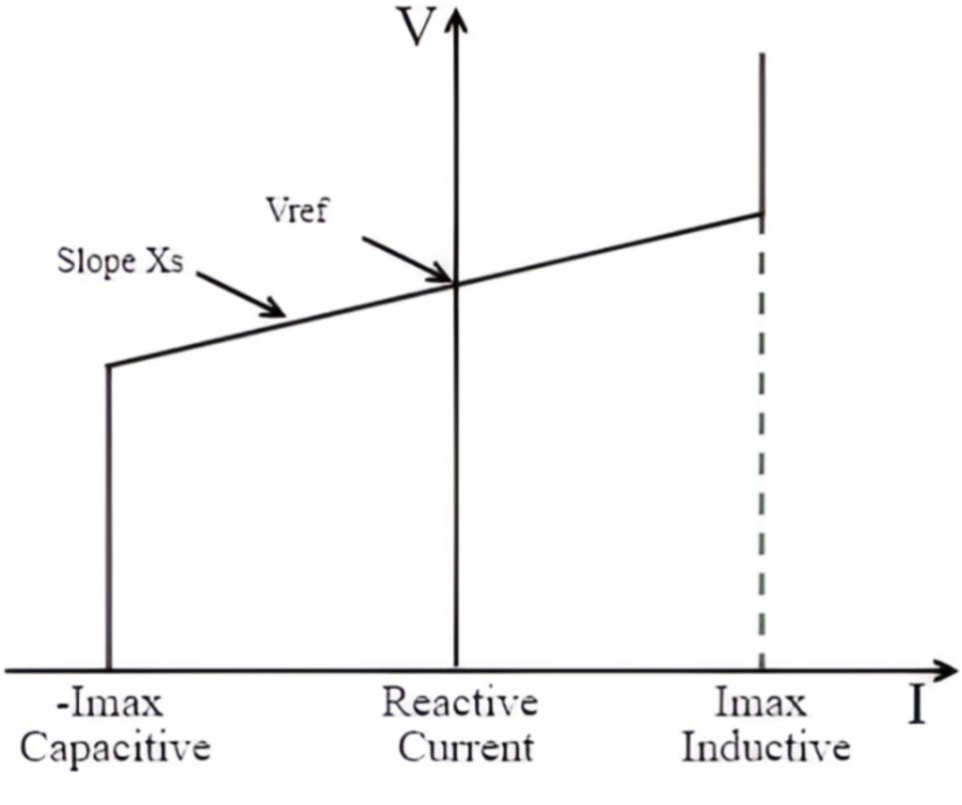
V-I characteristic of the turbine.

The control loop for the reactive power and voltage at the grid terminals is shown in Fig. 3. When the wind turbine is operating in voltage regulation mode, the voltage is changed to eliminate the error from the voltage of reference (Vref) as long as the reactive current stays within the maximum converter rating (-Imax, Imax). A voltage droop (Xs) is used, and Eq. (1) provides the slope of the V-I characteristic, which is shown in Fig. 4.

1 $$\:V={V}_{ref}+{X}_{S}I$$

B. Grid-Side Converter Controller.

**Fig. 5:**
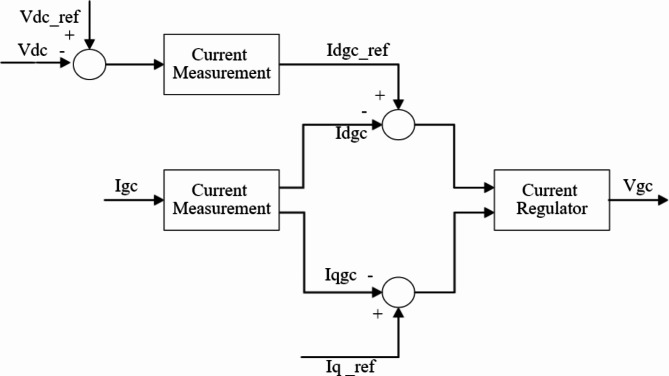
Grid-side converter controller.

It is the controller, which is installed on the grid side, and is used to manage the capacitor voltage of DC bus. The control loop is seen in Fig. 5^37^ and consists of:

- DC voltage regulator.
- Current regulator that regulates the grid-side converter’s generated voltage’s (V_gc_) phase and magnitude.

## Types of controllers

A. Enhanced PI Controller.

**Fig. 6:**
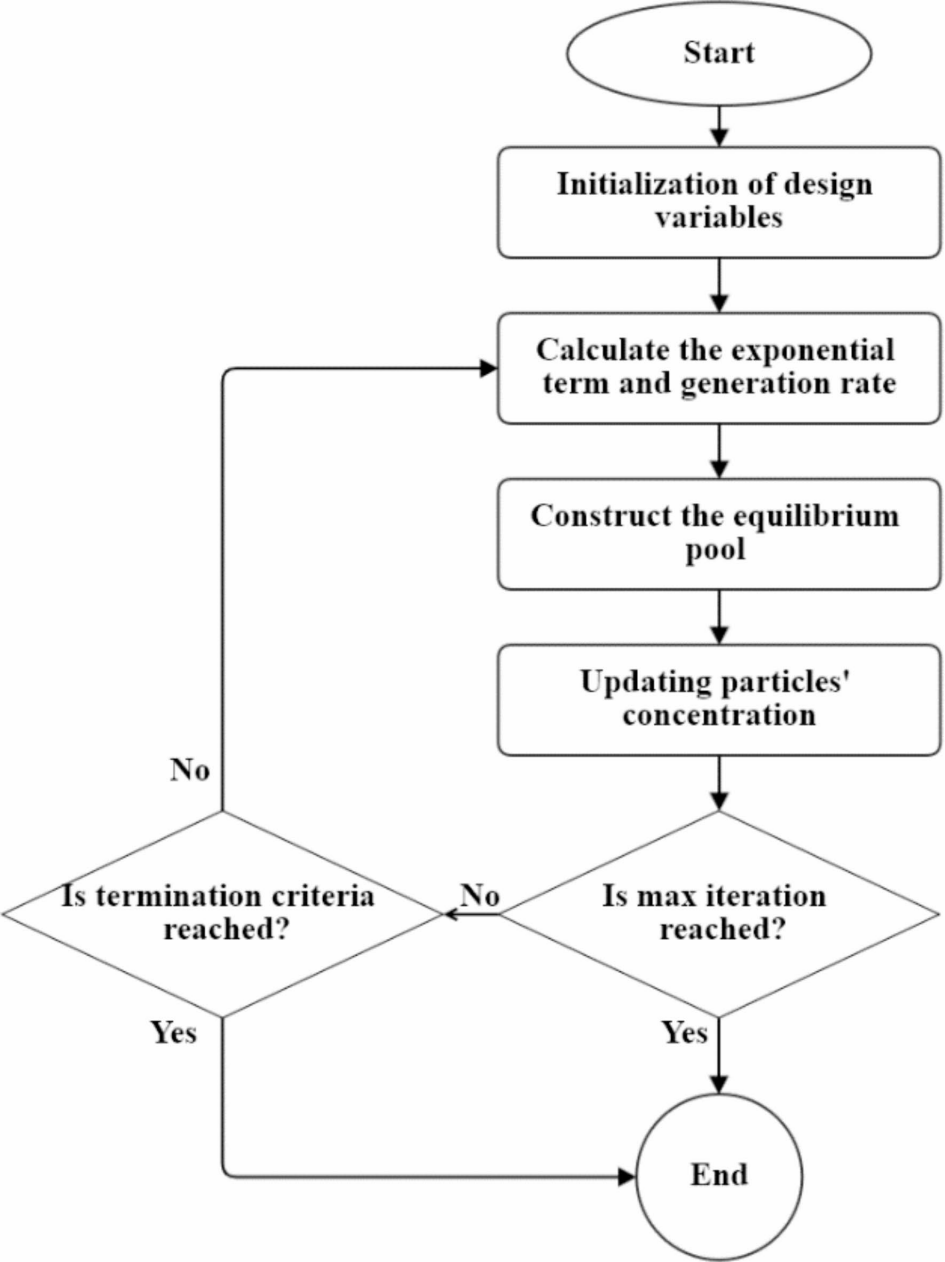
Equilibrium Optimization (EO) flow chart.

The enhanced PI controllers are used in the converters in the grid and rotor sides; The controllers gains are shown in Table 2have been optimized using equilibrium optimization (EO) algorithm which was influenced by the balanced mass models of the control volumes where the equation of the mass balance defines the mass of non-reactive components in a control volume by way of its source and sink processes^38,39^. Figure 6. depicts the flow chart of the EO algorithm.

**Table 2:** PI controllers’ parameters.

Controller parameters					
Converter in rotor side				Converter in grid side	
KP1	KI1	KP2	KI2	KP3	KI3
1.102	1.705	0.7622	125	0.0002	0.0005

B. Fuzzy Logic Controller.

Fuzzy Logic Control (FLC) mimics human reasoning by using tolerance, uncertainty, imprecision, and fuzziness in decision-making^31,40,41^and can provide a very satisfying performance without the requirement for an intricate mathematical model of the system^31,40,42^. Furthermore, FLC is more reliable and robust than conventional controllers, owing to its ability to handle operating conditions with a wide range^43^.

**Fig. 7:**
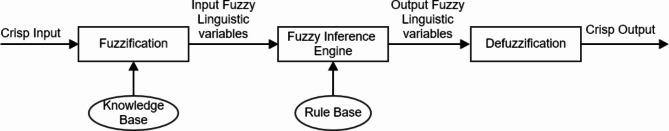
Structure of the FLC.

FLC is designed to mimic human deductive thinking^31^, which is the method humans use to draw conclusions from the information they have. Structure of FLC is shown in Fig. 7^29^.

The components of the FLC are^44^:

- Fuzzification: its job is to transform the values of crisp input into values of fuzzy.
- Knowledge Base: It has knowledge about all the membership functions of input and output.
- Rule Base: The If-Then rules where relationships between inputs and outputs are made.
- Fuzzy Inference Engine: it mimics human decisions by performing approximate thinking.
- Defuzzification: its job is to transform the fuzzy values into crisp values.

**Fig. 8:**
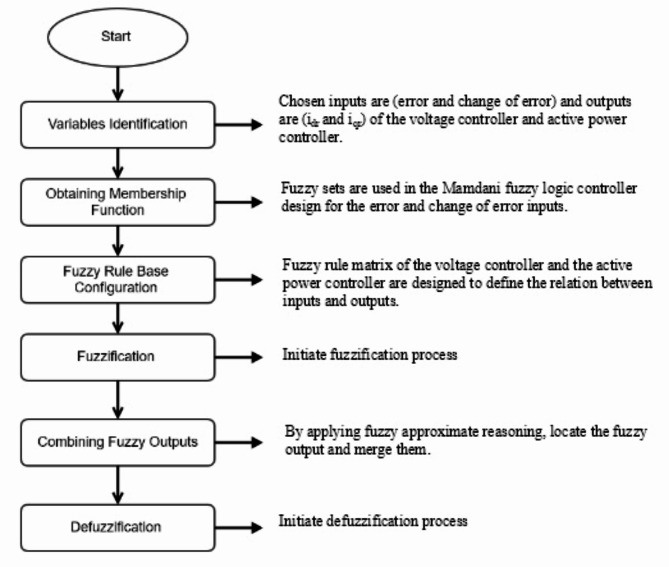
Steps of FLC design.

Steps in FLC design can be divided into six steps as illustrated in Fig. 8.

**Fig. 9:**
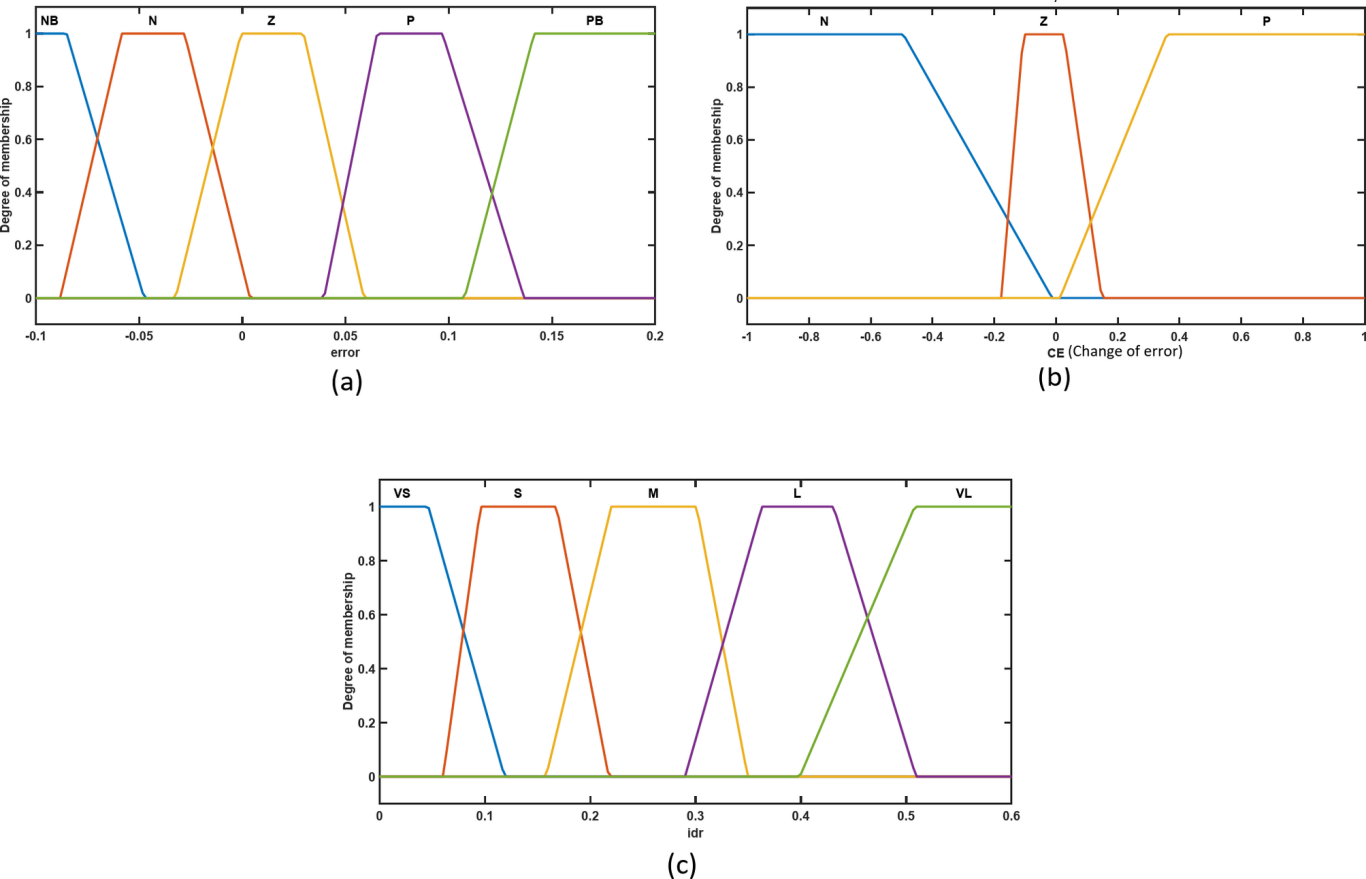
Trapezoidal membership function for voltage controller’s inputs and output; (**a**) Error membership function; (**b**) Change of error membership function; (**c**) i_qr_ membership function.

**Fig. 10:**
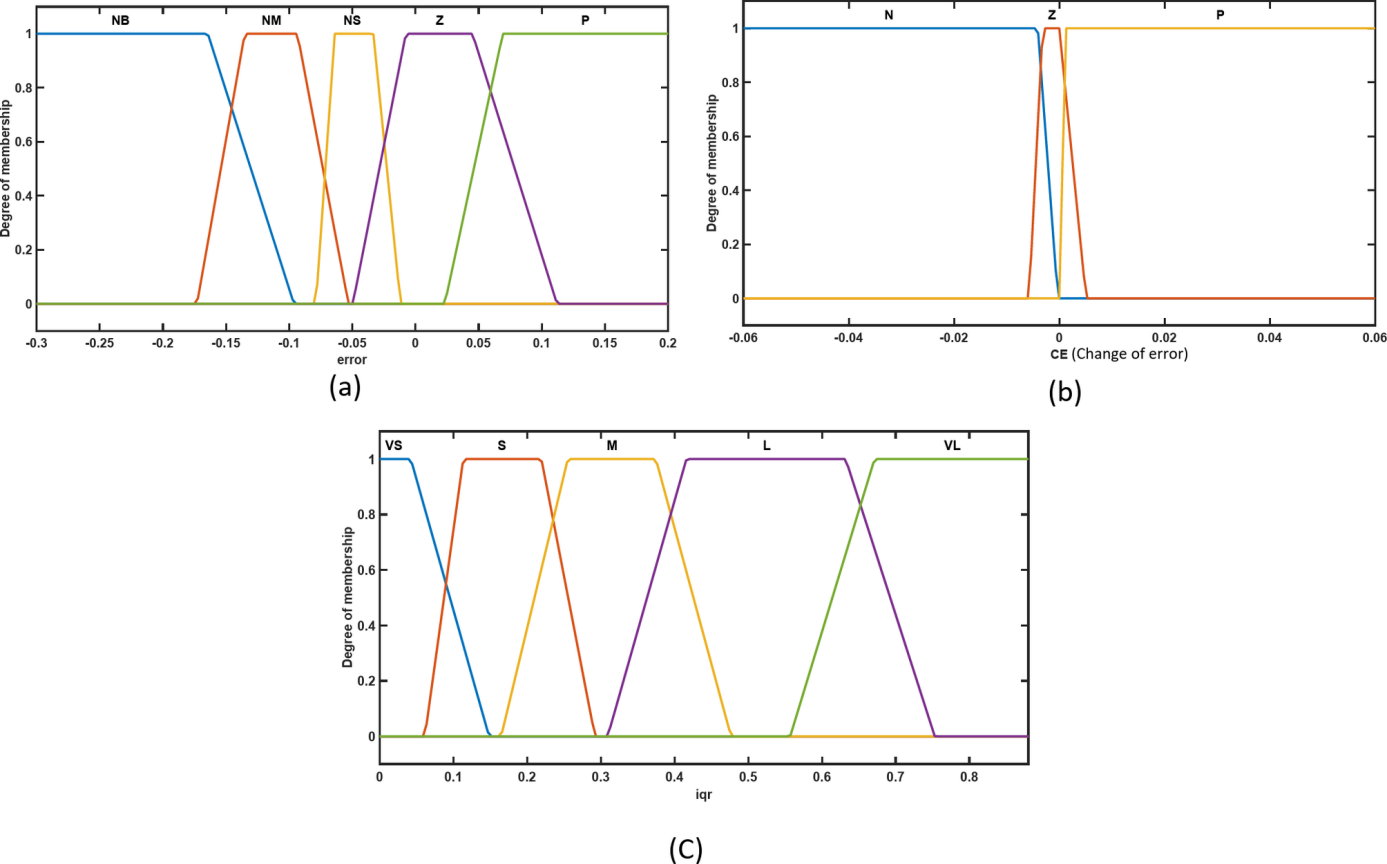
Trapezoidal membership function for active power controller’s inputs and output; (**a**) Error membership function; (**b**) Change of error membership function; (**c**) i_dr_ membership function.

The membership functions for the chosen inputs (error and change of error) and outputs (i_dr_ and i_qr_) of the voltage controller and active power controller are shown in Figs. 9 and 10 simultaneously. Five fuzzy sets are used in the Mamdani fuzzy logic controller design for the error input to transform and adapt the crisp form of the variables into their linguistic variable forms. These are the fuzzy sets: (i) NB (negative big), (ii) N (negative), (iii) Z (zero), (iv) P (positive), and (v) PB (positive big), For the change of error input, three fuzzy sets are used: (i) N (negative), (ii) Z (zero), and (iii) P (positive), and five sets are used for the outputs which are: (i) VS (very small), (ii) S (small), (iii) M (medium), (iv) L (large), and (v) VL (very large).

**Fig. 11:**
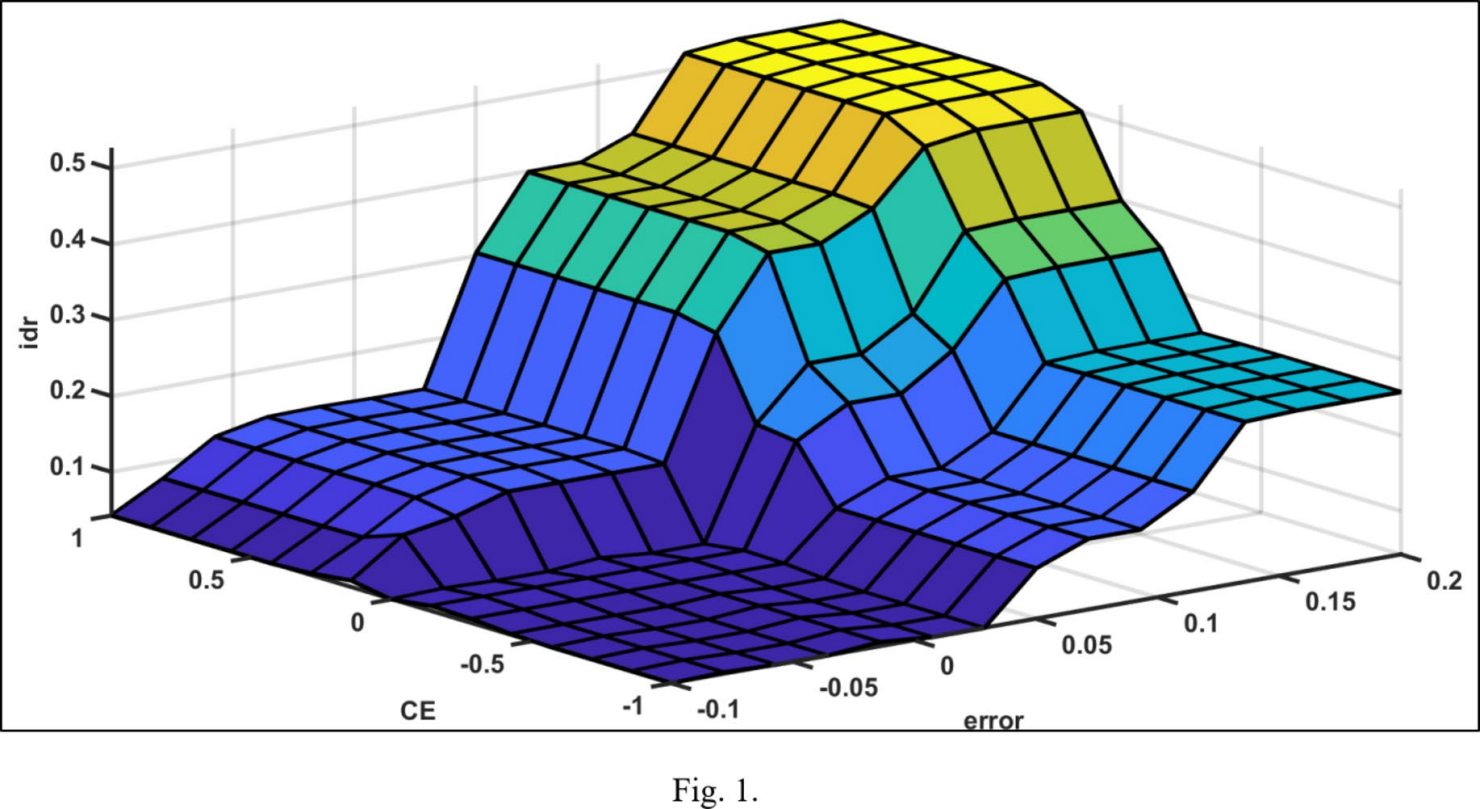
Fuzzy rule surface of the voltage controller.

**Fig. 12:**
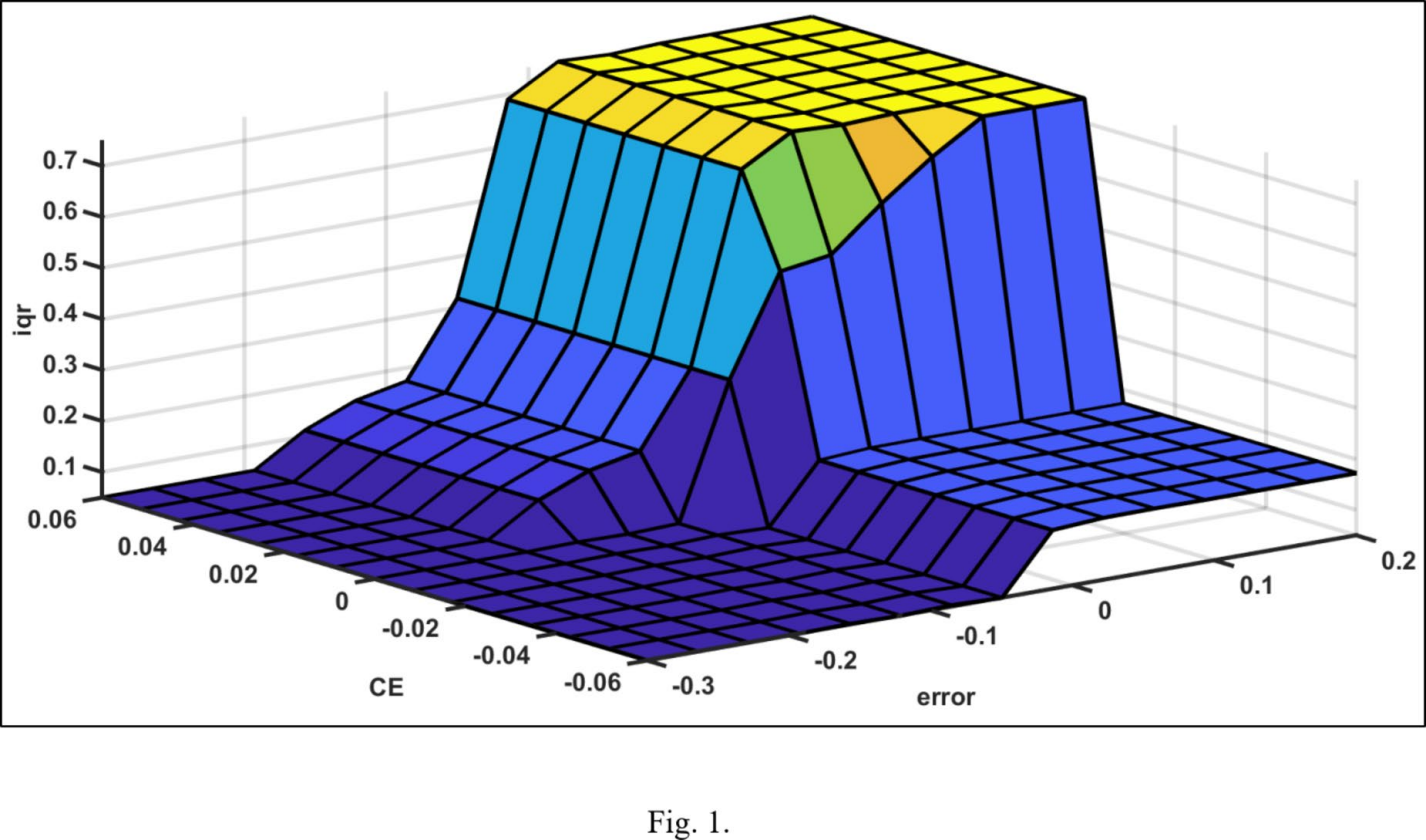
Fuzzy rule surface of the active power controller.

The rule matrix of the voltage controller and active power controller is shown in Tables 3 and 4 and illustrated In Figs. 11 and 12 respectively.

**Table 3:** Fuzzy rule matrix of the voltage controller.

E	NB	*N*	Z	*P*	PB
CE					
N	VS	VS	VS	S	M
Z	VS	VS	VS	M	L
P	VS	S	S	L	VL

**Table 4:** Fuzzy rule matrix of the active power controller.

E	NB	NM	NS	Z	*P*
CE					
N	VS	VS	VS	S	S
Z	VS	VS	M	L	VL
P	VS	S	M	VL	VL

## Simulation analysis

In this section the proposed controller is studied at two stages, first one is applying four distinct wind speed profiles, which correspond to the four seasons of the year, have been used to study the system. Twelve seconds of the prescribed fall wind speed are designated as the simulation time and compare the response of the proposed controller with the enhanced PI controller^1^.In second stage the proposed controller is examined against different types of faults to prove its reliability. The MATLAB/Simulink program is used to produce the simulation results.

### Applying four seasons wind profiles

**Fig. 13:**
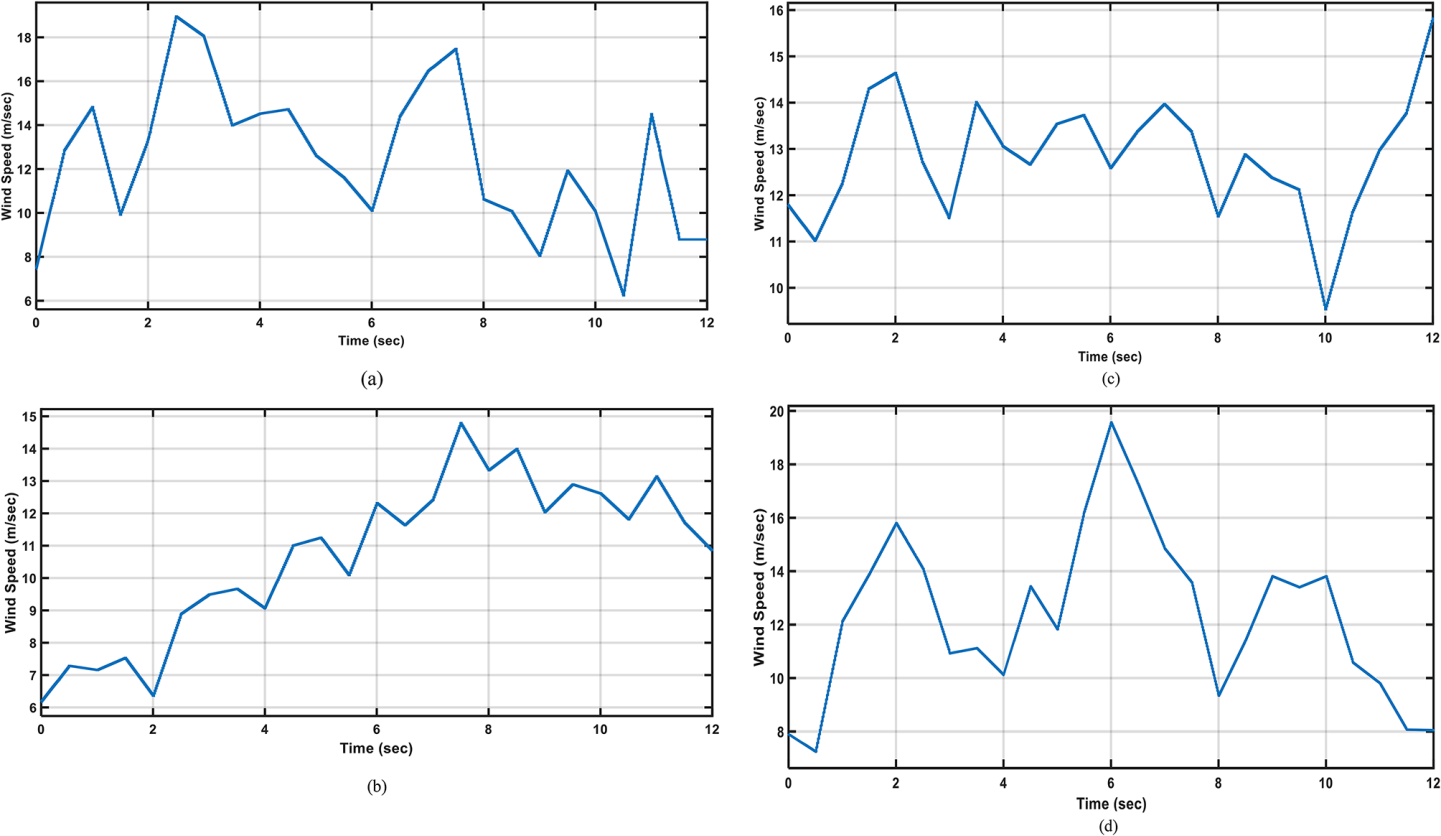
Different wind speed profiles; (**a**) April; (**b**) July; (**c**) October; (**d**) December.

**Fig. 14:**
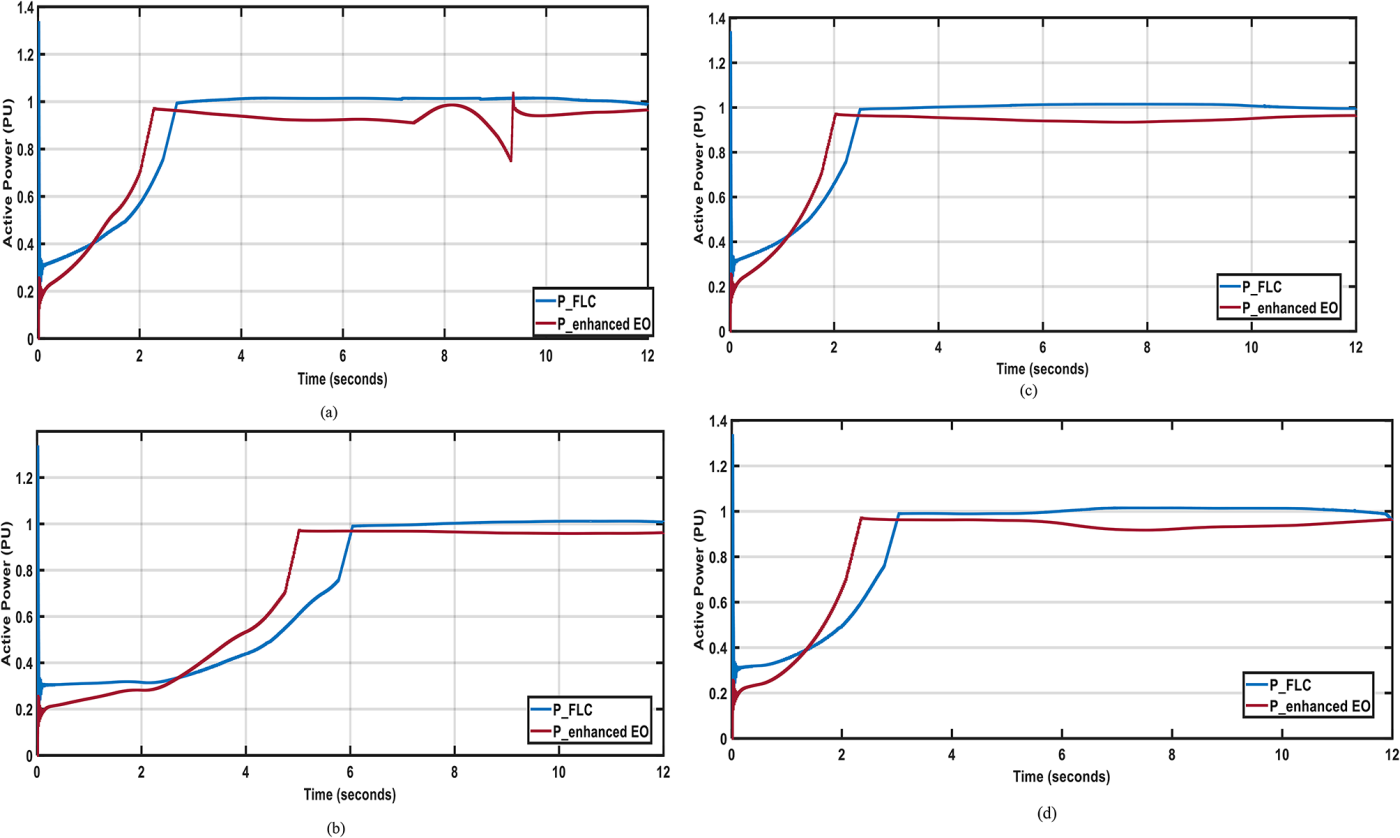
Output active power for different wind speed profiles; (**a**) April; (**b**) July; (**c**) October; (**d**) December.

In this section the wind speed profiles shown in Fig. 13^45^ are applied to the system, and Fig. 14shows the system output active power when utilizing the enhanced PI controller^1^ and the proposed FLC while the system is operating normally.

As depicted in Fig. 14(a), the enhanced PI controller^1^ shows oscillations and a poor response when subjected to April wind speed. These oscillations are a result of the sudden change in the speed of the wind at the seventh second. While the FLC shows better response to the change in the speed of the wind and lower steady state error. The enhanced PI controller and the FLC show almost the same good response when subjected to July, October, and December wind speed profiles, as depicted in Fig. 14 (b, c, and d), respectively, with the FLC having an advantage due to the lower steady state error. Moreover, The FLC has better Settling time by around 66%,14%,69% nd 70% nd better steady state error by around 2% 4%,4% nd 2% han enhanced PI in April, July, October; and December respectively.

### Robustness analysis

The system is studied under different operating conditions to study the robustness of the FLC which shows its superiority over the enhanced PI controller^1^ under the system operating in normal conditions. Therefore, severe fault conditions (one line to ground fault, two lines to ground fault, and three line to ground fault) have been subjected to the system; the fault occurred at 12.5 km from the first step-up voltage transformer. At t = 3, the disturbance—which has a period of 1 s—represents a momentary fault. When the FLC and the optimized PI controller are used, the output active powers are compared for systems that are subjected to various fault conditions and wind speed profiles.

A. One-Line to Ground Fault.

**Fig. 15:**
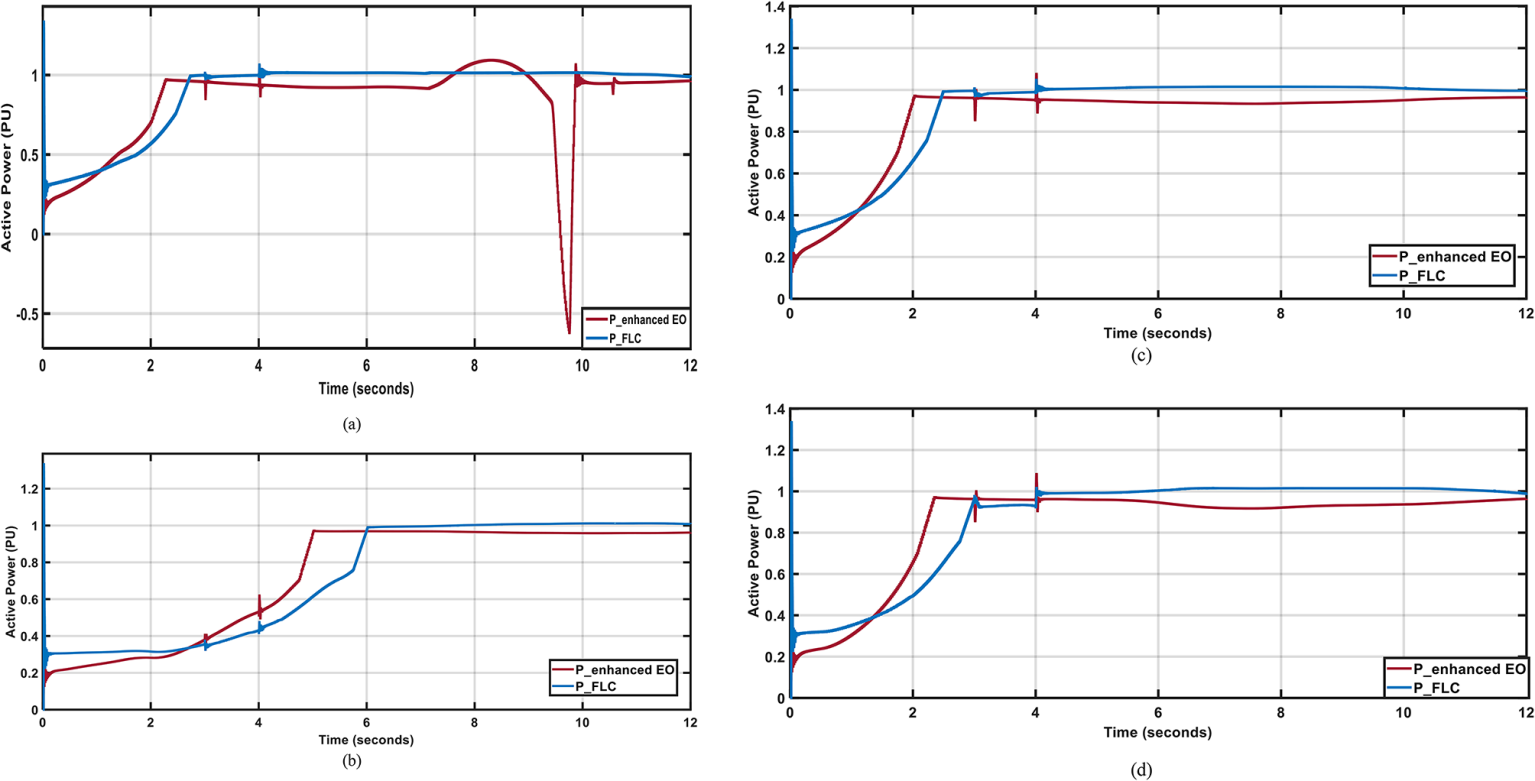
Output Active Powers under 1-LG fault for different wind profiles; (**a**) April; (**b**) July; (**c**) October; (**d**) December.

Figure 15(a) demonstrates that FLC has better response and low steady state errors during the fault time when compared with the PI controller, which shows poor performance during the fault time and many oscillations at the seventh second as well. The enhanced PI controller^1^ and the FLC show the same results when applying July, October, and December profiles, as shown in Fig. 15 (b, c, and d), respectively, with the FLC having an advantage due to the lower steady state error and the better performance during the fault time with sustaining the power to 1 PU. Moreover, The FLC has better Settling time by around 70%,40%,60% nd 65% nd better steady state error by around 2% 4%,2% nd 2% han enhanced PI in April, July, October; and December respectively.

B. Two-Line to Ground Fault.

**Fig. 16:**
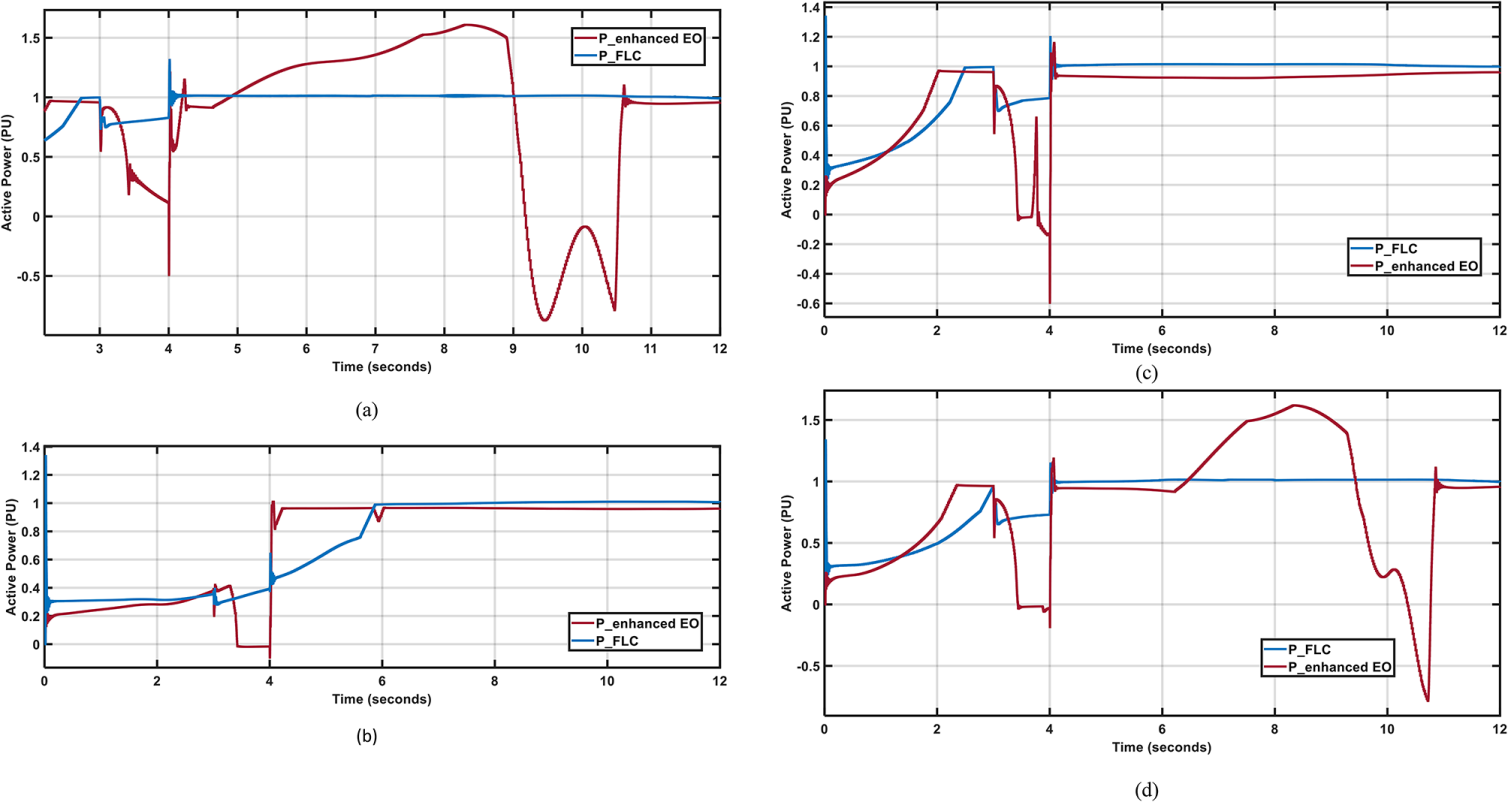
Output Active Powers under 2-LG fault for different wind profiles; (**a**) April; (**b**) July; (**c**) October; (**d**) December.

Figure 16(a and d) show that the FLC has good response during the fault with lower oscillations and minimal steady state error after the fault clearance, while the enhanced PI controller^1^ shows poor response during the fault time with no ability to keep the power above zero PU and shows huge oscillations after the fault clearance. Comparing the FLC to the PI controller, Fig. 16(b and c) shows that the FLC has substantially greater response during the fault time and negligible steady state error. Moreover, The FLC has better settling time 64%,14%,60% nd 63% han enhanced PI in April, July, October; and December respectively. Also, The FLC has better peak to peak oscillations 60%,50%,30% nd 50% han enhanced PI in April, July, October; and December respectively.

C. Three-Line to Ground Fault.

**Fig. 17:**
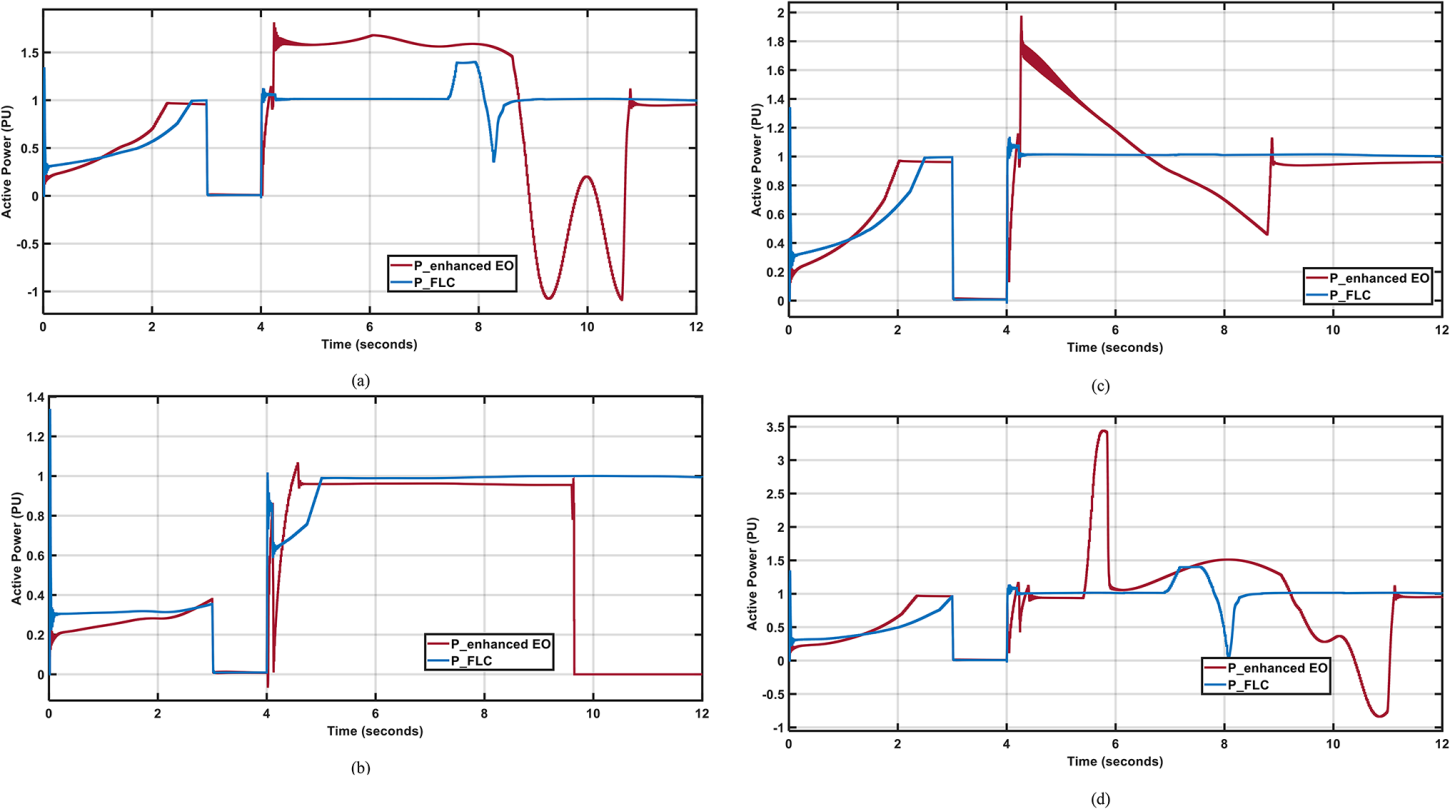
Output Active Powers under 3-LG fault for different wind profiles; (**a**) April; (**b**) July; (**c**) October; (**d**) December.

Figure 17 (a, c, and d) show that the FLC has minimal steady state error and better response after the fault clearance compared to the PI controller which has much higher max overshot and higher oscillations. Figure 17(b) shows the superiority of the FLC compared to the enhanced PI controller^1^ when subjected to this severe three line to ground fault as it succeded to sustain the system to 1 PU after fault clearance while the PI controller could not return to the normal operating point 1 PU. Moreover, The FLC has better settling time 64%, 4%, 0% ad 63% tan enhanced PI in April, July, October; and December respectively. Also, The FLC has better peak to peak oscillations 33%, 0%, 0% ad 65% tan enhanced PI in April, July, October; and December respectively.

## Conclusion

This paper contains detailed case studies for grid connected DFIG and the installed control strategies to regulate the output active power. Performance of an enhanced PI controller, which is optimized by using EO algorithm, is compared with FLC. The FLC shows its superiority in providing wide scope of control under rapid variations in wind speed with better response and minimal steady state error. A robustness analysis has been performed to ensure the FLC performance when subjected to severe operating conditions (1-LG, 2-LG, and 3-LG) faults. The FLC shows better response when applying different wind profiles and different fault conditions because of the better response, lower oscillations, and lower steady state error. The FLC demonstrates a better settling time compared to the enhanced PI, with improvements ranging from approximately 14–70% under normal conditions and 40–70% under various fault conditions. Furthermore, the FLC exhibits lower peak-to-peak oscillations than the enhanced PI in fault conditions, with reductions of about 30–65%. Additionally, the FLC achieves a smaller steady-state error compared to the enhanced PI, with improvements of around 2–4% under normal conditions and most fault scenarios. In future works, the output voltage, the DC link voltage, and the reactive power can also be investigated to improve the DFIG performance using the proposed controllers. Further, different real time controllers can be discussed to achieve grid requirements for the power quality.

## Data Availability

The datasets used and/or analyzed during the current study are available from the corresponding author on reasonable request.
